# Sertraline Delivered in Phosphatidylserine Liposomes Is Effective in an Experimental Model of Visceral Leishmaniasis

**DOI:** 10.3389/fcimb.2019.00353

**Published:** 2019-10-29

**Authors:** Maiara Maria Romanelli, Thais Alves da Costa-Silva, Edezio Cunha-Junior, Daiane Dias Ferreira, Juliana M. Guerra, Andres Jimenez Galisteo, Erika Gracielle Pinto, Leandro R. S. Barbosa, Eduardo Caio Torres-Santos, Andre Gustavo Tempone

**Affiliations:** ^1^Centre for Parasitology and Mycology, Instituto Adolfo Lutz, São Paulo, Brazil; ^2^Fundação Oswaldo Cruz, Instituto Oswaldo Cruz, Pavilhão Leonidas Deane, Laboratório de Bioquímica de Tripanosomatídeos, Rio de Janeiro, Brazil; ^3^Centre for Pathology, Instituto Adolfo Lutz, São Paulo, Brazil; ^4^Faculdade de Medicina, Hospital das Clínicas HCFMUSP, Universidade de São Paulo, São Paulo, Brazil; ^5^Drug Discovery Unit, Life Sciences, University of Dundee, Dundee, Scotland; ^6^Instituto de Física da Universidade de São Paulo, Cidade Universitária, São Paulo, Brazil

**Keywords:** leishmania, neglected diseases, drug delivery, liposomes, drug repurposing, sertraline

## Abstract

Liposomes containing phosphatidylserine (PS) has been used for the delivery of drugs into the intramacrophage milieu. *Leishmania* (L.) *infantum* parasites live inside macrophages and cause a fatal and neglected viscerotropic disease, with a toxic treatment. Sertraline was studied as a free formulation (SERT) and also entrapped into phosphatidylserine liposomes (LP-SERT) against intracellular amastigotes and in a murine model of visceral leishmaniasis. LP-SERT showed a potent activity against intracellular amastigotes with an EC_50_ value of 2.5 μM. The *in vivo* efficacy of SERT demonstrated a therapeutic failure. However, when entrapped into negatively charged liposomes (−58 mV) of 125 nm, it significantly reduced the parasite burden in the mice liver by 89% at 1 mg/kg, reducing the serum levels of the cytokine IL-6 and upregulating the levels of the chemokine MCP-1. Histopathological studies demonstrated the presence of an inflammatory infiltrate with the development of granulomas in the liver, suggesting the resolution of the infection in the treated group. Delivery studies showed fluorescent-labeled LP-SERT in the liver and spleen of mice even after 48 h of administration. This study demonstrates the efficacy of PS liposomes containing sertraline in experimental VL. Considering the urgent need for VL treatments, the repurposing approach of SERT could be a promising alternative.

## Introduction

Leishmaniasis is a neglected infectious disease caused by an intracellular protozoan known as *Leishmania* spp. Visceral leishmaniasis (VL) is highly endemic in the South America, where it is caused by *Leishmania* (L.) *infantum*; in the Indian subcontinent; and in the east of Africa, where the etiologic agent is *Leishmania* (L.) *donovani*; the WHO (World Health Organization, 2017) estimates more than 400,000 new cases of VL every year. More than 90% of these cases are concentrated in Bangladesh, Brazil, Ethiopia, India, Sudan, and Southern Sudan (Alvar et al., 2012; World Health Organization, 2017). VL is a chronic and systemic disease affecting liver, spleen, and bone marrow, and if not adequately treated, it results in 100% of death. The current therapy is challenging due to a limited number of available drugs, elevated costs due to hospitalization, high toxicity, parenteral administration, and the emergence of resistance to conventional drugs (Alvar et al., 2012). Limited options are available for the treatment of VL. In the Indian subcontinent, a single dose of AmBisome and combination therapy are the preferred treatment options. The combination of antimony with paromomycin is the choice in East Africa and Yemen. In the Mediterranean Basin, Middle East, and Central Asia, AmBisome remains the main choice. According to the recent PAHO guidelines, AmBisome, SbV, and conventional AmB are still the recommended drugs for the treatment of VL in the New World (Chakravarty and Sundar, 2019). Among candidate drugs for VL is paromomycin, an aminoglycoside antimicrobial, which showed efficacy as a parenteral drug and entered for Phase III in 2005 (Croft et al., 2005). Considering the lack of therapeutic options for the treatment of VL, the need for novel drugs is evident (Tempone et al., 2017).

Finding new uses for FDA-approved drugs is known as drug repositioning, and has been considered an excellent approach for neglected diseases to reduce the costs and time of the research (Andrews et al., 2014; Huang et al., 2015; Costa-Silva et al., 2017). Currently, all available drugs for the clinical treatment of leishmaniases were introduced by the repositioning approach. Pentamidine, an aromatic diamine, was first synthesized by May and Baker Co, during the preparation of antitrypanosomal (*Trypanosoma brucei*) compounds (Lourie and Yorke, 1939). The first report describing the activity of amphotericin B against *Leishmania* was in 1960 (Furtado et al., 1960). However, amphotericin B was initially licensed in 1959 for the treatment of progressive and potentially life-threatening fungal infections (Ostrosky-Zeichner et al., 2003). Miltefosine (hexadecylphosphocholine) was synthesized as part of an anti-inflammatory program in 1982 at the pharmaceutical company Burroughs Wellcome (USA) (Croft and Engel, 2006). A series of alkyl phospholipids analogs made by Takeda Co. demonstrated effective *in vitro* properties as antifungals (Tsushima et al., 1982), but only 2 years later, these compounds were selected for screening against *Leishmania* and trypanosomes at the Wellcome Research Laboratories (UK). Finally, paromomycin, an oral broad-spectrum aminoglycoside antibiotic synthesized in 1959 by Carlo Erba Co. (Botero, 1978), was studied as an antileishmanial candidate in 1975 (Mattock and Peters, 1975).

Drugs approved for central nervous system like antidepressants are usually safe and widely used worldwide. Antidepressants and tricyclic neuroleptic drugs have shown antileishmanial activity (Evans and Croft, 1994; Chan et al., 1998; Richardson et al., 2009). Another widely used antidepressant, imipramine, showed potential antileishmanial effect (Andrade-Neto et al., 2016), with promising *in vivo* efficacy (Mukherjee et al., 2014). Additionally, imipramine has shown to depolarize the transmembrane mitochondrial potential of *L*. (L.) *donovani* (Mukherjee et al., 2012) and altered the sterol level of *Leishmania* (L.) *amazonensis* (Andrade-Neto et al., 2016). In this context, sertraline (SERT), a selective serotonin reuptake inhibitor (SSRI), presents several therapeutic uses, ranging from management of depression, to control of obsessive–compulsive disorder and social phobia, to treatment of chronic pain (Kreilgaard et al., 2008; Santuzzi et al., 2012). Palit and Ali (2008) demonstrated the activity of SERT against the Indian etiologic agent of VL, *L*. (L.) *donovani*, and demonstrated both *in vitro* and *in vivo* efficacy at elevated doses.

The lethal action of sertraline was also investigated in *L. infantum* parasites. The drug induced respiration uncoupling, with a significant decrease of intracellular ATP level, and also induced oxidative stress in *Leishmania*. Metabolomics data demonstrated an extended metabolic disarray caused by sertraline, with a remarkable variation of the levels of thiol-redox and polyamine biosynthetic intermediates, suggesting a multitarget mechanism of action (Lima et al., 2018).

Drug delivery systems can direct antileishmanial substances to infected organs (Carvalheiro et al., 2015). Negatively charged nanoliposomes containing pentavalent antimony has shown superior efficacy than free pentavalent antimony for experimental VL treatment due to the targeting ability of these vesicles to bind host cell receptors, named scavenger receptors (ScavR) (Tempone et al., 2004).

In this work, we evaluated, for the first time, the *in vivo* efficacy of the antidepressant sertraline entrapped into negatively charged liposomes (LP-SERT) in a VL-experimental murine model and studied its immunomodulatory effect after treatment. Additionally, a delivery assay was developed to demonstrate the targeting ability of LP-SERT to spleen and liver organs. *In vitro* studies were also performed to evaluate host cell uptake, mammalian cytotoxicity, and *in vitro* efficacy.

## Materials and Methods

### Drugs and Chemicals

3-[4,5-Dimethylthiazol-2-yl]-2,5-diphenyltetrazolium bromide (thiazol blue; MTT), sodium dodecyl sulfate (SDS), M-199 medium, RPMI-PR-1640 medium (w/o phenol red), and cholesterol were purchased from Sigma–Aldrich (St. Louis, MO, USA). Hydrogenated phospholipids were kindly donated by Lipoid GmbH (Ludwigshafen, Germany). Sertraline and other analytical reagents were purchased from Sigma–Aldrich (St. Louis, MO, USA). Molecular biology reagents are purchased from Life Technologies, and CBA (cytometric beads array) was purchased from BD (San Jose, CA, USA).

### Parasites and Macrophages

*L*. (L.) *infantum* (MHOM/MA67ITMAP263) amastigotes were maintained by using promastigotes from the culture that were isolated from the liver of previously infected mice. The animals were infected with 1 × 10^8^ amastigotes (100 μL) by intra-peritoneal route. After 15 days post infection (d.p.i.), the animals were euthanized and the liver was macerated in a tissue grinder tube containing 5 ml of PBS and the amastigotes were separated by differential centrifugation to obtain a suspension of parasites. Promastigotes were maintained in Schneider's medium supplemented with 10% fetal bovine serum (FBS) at 24°C. Macrophages were collected from the peritoneal cavity of BALB/c mice by washing with RPMI-1640 medium supplemented with 10% FBS and were maintained in a 5% CO_2_-humidified incubator at 37°C (Cunha-Júnior et al., 2016).

### Experimental Animals

BALB/c mice were obtained from the Instituto Adolfo Lutz of São Paulo State–Brazil, kept in sterile boxes with absorbent material, and received food and water *ad libitum*. BALB/c mice were infected each month with promastigotes from the culture to maintain the *Leishmania* strain. BALB/c mice were also used to obtain peritoneal macrophages. Animal experiments were performed with the approval of the Ethics Committee of Instituto Adolfo Lutz (project CEUA-IAL/Pasteur 04/2016) in accordance with the National Institutes of Health Guide for the Care and Use of Laboratory Animals (NIH Publications No. 8023).

### Determination of 50% Effective Concentration (EC_50_) of SERT and LP-SERT in Promastigotes and Amastigotes of *L*. (L.) *infantum*

To determine the 50% inhibitory concentration against promastigotes (final logarithmic phase of growth), SERT was dissolved in DMSO, and the standard drug (miltefosine) was dissolved in Mili-Q water; both drugs were diluted in Schneider's medium in 96-well microplates with an initial concentration of 100 μM. The promastigotes were counted in a Neubauer chamber and seeded at 1 × 10^6^/well at the final volume of 150 μl. Controls with DMSO and without drugs were performed. The plate was incubated for 48 h, and the viability of promastigotes was determined by MTT assay. Promastigotes incubated without the drug was used as the viability control. The optical density was determined using a plate reader FilterMax at 570 nm and the data analysis was performed using GraphPad Prism 5.0 software (Mikus and Steverding, 2000).

To determine the EC_50_ value for SERT and LP-SERT against *L*. (L.) *infantum* intracellular amastigotes, peritoneal macrophages (1 × 10^5^/well) were collected from the peritoneal cavity of BALB/c mice added to 16-well chamber slides (NUNC) and incubated for 24 h at 5% CO_2_ and 37°C. A two-step washing procedure was performed to eliminate non-adherent cells. Promastigotes were added at a ratio of 10:1, using RPMI 1640 supplemented with 2% horse serum (Rebello et al., 2019), and after 4 h, the extracellular parasites were removed by washing; fresh medium containing SERT, LP-SERT, and control were added; and the cells were incubated at 37°C for 72 h. The initial concentration of SERT and miltefosine was 100 μM, and that for LP-SERT was 20 μM. At the end of the assay, the slides were stained with Giemsa and observed using a light microscopy. The EC_50_ was determined by the number of infected macrophages in 400 cells (Yardley and Croft, 2000).

### Determination of Cytotoxicity of SERT and LP-SERT in Mammalian Cells

NCTC cells were seeded at 6 × 10^4^ cells/well in 96-well microplates and incubated with SERT, LP-SERT, and miltefosine with 150 μM as the highest concentration, for 72 h at 37°C in a 5% CO_2_ humidified incubator. The viability of the cells was determined by the MTT assay. Control cells were incubated in the presence of DMSO and without drugs. Viability of 100% was expressed based on the optical density of control NCTC cells, after normalization. The selectivity index (SI) was given by the ratio between the cytotoxicity in NCTC cells and the anti-parasitic activity of the drugs (Pinto et al., 2014).

### *In vitro* Uptake of LP-SERT by Macrophages

Macrophages were obtained from the peritoneal cavity of BALB/c mice and seeded at 5 × 10^6^/well for 24 h in 24-well plates. The cells were infected with promastigotes at a ratio of 1:10 (macrophage:parasites). After 4 h of infection, infected and uninfected cells were treated with rhodamine 123-labeled liposome (LP-SERT-R123) (10 μg/ml). The cells were scraped from their wells using a sterile cell scraper and the fluorescence intensity was measured by flow cytometry (Attune-Life technologies) up to 270 min. The excitation and emission wavelengths were 488 and 575 nm, respectively. A number of 10,000 events per sample/time were evaluated to observe the internalization of labeled liposomes. Untreated cells and liposomes without drug were used as controls.

### SERT Entrapment in Liposomes

Encapsulation of SERT was performed as described by Reimão et al. (2012). Briefly, for the liposome preparation, 6 mg of sertraline was dissolved in methanol and sonicated in a bath sonicator for 10 min at 25°C (solution A). Solution B consisted of saturated egg phosphatidylcholine (25 mg), saturated egg phosphatidylserine (7.62 mg), and cholesterol (1.81 mg) at 7:2:1 mole ratio, dissolved in 1 ml of chloroform:methanol (1:1). The mixture of solutions A and B was further sonicated for 10 min. The mixture was evaporated in a rotary evaporator at 55°C for 40 min in a vacuum and protected from light. A pre-heated (55°C) isotonic solution of 2.25% glycerol (1 ml, v/v) was added to the lipid film using glass beads. The swelling process of the pre-formed liposomes was performed in a rotary evaporator at 55°C for 60 min without vacuum. The liposomes were sonicated in a bath sonicator under heating (55°C) for 30 min, followed by extrusion in membranes of 0.8, 0.4, and 0.2 nm. The untrapped SERT was separated from the liposomes by centrifugation (4,000 *g* for 15 min) (Reimão et al., 2012).

### Quantification of SERT Into Liposomes

The concentration of the encapsulated SERT was determined using an ultra-high-performance liquid chromatography (UPLC) with a binary AT system (Prominence LC-20; Shimadzu Corp., Kyoto, Japan) and an ultraviolet photodiode detector array (PDA) SPDM20A on a reverse-phase ACE C4 column (4.6 mm × 250 mm, 5 μm particle size). The wavelength was set at 220 nm. The flow rate was 1 ml/minute using an isocratic method with acetonitrile:methanol (7:3 v/v) and 0.1% trifluoroacetic acid (TFA). The SERT was diluted in isopropanol (1 mg/ml) to obtain standard solutions in a range of 3.125–400 μg/ml, and 20 μl samples were injected into the column. Results obtained were extrapolated from a standard curve using a linear regression curve. The encapsulation efficiency (%EE) was calculated using the following equation (Stewart, 1980; Ong et al., 2016). Free SERT is considered the non-entrapped drug. Liposomes were used in all assays immediately after preparation.

$$\begin{array}{r} \%EE\;=\;\left(total\;SERT-free\;SERT\right)/\left(total\;SERT\right)\times \;100 \end{array}$$

### Dynamic Light Scattering (DLS)

DLS measurements were applied to obtain the vesicles' mean diameter in the absence and presence of SERT, through the apparent diffusion coefficient measurement. The DLS was performed in a ZetaSizer ZS90 equipment (Malvern, UK) using the detector positioned at 90° and a He–Ne laser, λ = 632.8 nm, as a light source. All measurements were performed at 22°C. Samples were diluted 20-fold to ensure no multiple scattering inside the cuvette during the experiments. The results show a unimodal distribution and represent the average of, at least, four experiments.

The apparent values of hydrodynamic diameter, which is related to the diffusional dynamics of a vesicle, were obtained with the autocorrelation functions and were analyzed by the cumulant analysis software provided by Malvern. The *D*_*h*_ determination was calculated by means of Stokes–Einstein, as the following equation:

$$\begin{array}{l} D_{h}=\frac{k_{B}T}{3\pi \eta D_{app}} \end{array}$$

where *k*_*B*_ is the Boltzmann constant, *T* is the absolute temperature, η is the viscosity of the solvent, and *D*_*h*_ and *D*_*app*_ are the hydrodynamic diameter and the apparent diffusion coefficient, respectively (Costa-Silva et al., 2017).

### ζ-Potential

ζ-Potential measurements of PS-containing vesicles in the absence and presence of SERT were evaluated in order to check the effect of the drug on the vesicle effective surface charge. The ζ-potential was performed in a ZetaSizer ZS90 equipment (Malvern, UK) using the detector positioned at 173°, a He–Ne laser, λ = 632.8 nm, as a light source and applying PALS (phase analysis light scattering) and LDV (Laser Doppler Velocity) to calculate the electrophoretic mobility and the ζ-potential. The ζ-potential is a measure of the system stability, since it is able to give information about the net change at the vesicle surface. Actually, the ζ-potential is defined as the electrical potential in the beginning of the so-called double layer, i.e., the slipping plane of the vesicle surface. In this study, the ζ-potential was obtained using the electrophoretic mobility, based on the Helmholtz–Smoluchowski equation, which is used in water-based systems in the presence of ionic strength (*I*) > 1 mM and particle diameter (*D*_H_), > 100 nm. The measurement for each sample was repeated for at least three times. In order to avoid multiple scattering, the samples were diluted 20-fold in the appropriate buffer before the measurements (Lamy et al., 1966; Costa-Silva et al., 2017). LP-SERT treatment was analyzed by culture microtitration as previously reported (Park and Lee, 2008).

### Experimental Assay

#### Dose Translation

The initial dose was calculated based on the human dose, as follows:

$$\begin{array}{r} Animal\;dose\;\left(mg/kg\right)=\left(\frac{human\;Km}{animal\;km}\right)\times \;human\;dose\; \end{array}$$

where mouse Km = 3, human Km = 37 (Reagan-Shaw et al., 2008).

#### Determination of the Efficacy of SERT in L. (L.) Infantum-Infected Mice

Young female BALB/c mice were previously infected with *L*. (L.) *infantum* at 1 × 10^8^ parasites/animal by intra-peritoneal route. The animals were treated at the 5th d.p.i. by the oral route for 10 consecutive days as follows: Group 1—untreated group; Group 2—SERT at 0.3 mg/kg/day; Group 3—SERT at 1 mg/kg/day. Each group consisted of five animals (*n* = 5/group). The animals were euthanized at the 16th d.p.i. and the parasitic load was analyzed by culture microtitration analysis (Buffet et al., 1995). Sertraline was dissolved and diluted in saline solution (0.9% m/v).

#### Determination of the Efficacy of LP-SERT in L. (L.) Infantum-Infected Mice

Young female BALB/c mice were previously infected with *L*. (L.) *infantum* at 1 × 10^8^ parasites/animal by intra-peritoneal route. The animals were treated at the 5th d.p.i. by the subcutaneous route for 10 consecutive days as follows. Group 1—untreated group; Group 2—LP-SERT at 0.3 mg/kg/day; Group 3—LP-SERT at 1 mg/kg/day; Group 4—treated group with liposome in the absence of the drug. Each group consisted of five animals (*n* = 5/group). The animals were euthanized at the 16th d.p.i. and the parasitic load was analyzed by culture microtitration analysis (Buffet et al., 1995). As a control of this experiment, a study was performed using the free drug in the subcutaneous route in the same doses. Liposomal sertraline was diluted in isotonic glycerol solution (2.2% v/v).

### Immunomodulatory Studies

Based on the results of the treatment with LP-SERT in *L*. (L.) *infantum*-infected mice, the group treated at 1 mg/kg/day was chosen for immunomodulatory studies. The serum cytokines were analyzed by flow cytometry using the CBA kit. The CBA kit quantifies the cytokines (IL-4, INF-γ, TNF-α, IL-6, IL-10, and IL-12) and chemokine MCP-1 involved in the inflammatory response (Costa-Silva et al., 2017). Six populations of beads with different intensities of fluorescence light conjugate with antibody of capture for each cytokine were mixed, and after the incubation with the serum, the samples were analyzed by flow cytometry (LSR Fortessa).

### Histopathology Profile

After the treatment with LP-SERT, the animals were euthanized and the necropsy was performed with macroscopic and microscopic evaluation of the organs (liver and spleen). Fragments of organs were immediately removed, preserved in 10% buffered formalin, and processed by usual histological techniques. After these procedures, the slides were stained with hematoxylin and eosin technique. The analyses were performed in light microscopy to characterize and describe histopathological lesions (Guerra et al., 2016).

### Targeting of LP-SERT to Spleen and Liver

To confirm the targetability of LP-SERT to spleen and liver of animals, BALB/c mice were infected with 1 × 10^8^ promastigotes of *L*. (L.) *infantum*; after 10 days of infection, the animals were treated (one dose) with LP-SERT labeled with fluorescent probe DIL C18. After 24 h of treatment, the animals were euthanized. The liver and spleen were removed, and imprint of the organs was done in glass slides, fixed with 3.5% formaldehyde for 6 min, and washed with phosphate buffered saline (PBS). The PGN solution (PBS, 0.2% gelatin and 0.15% NaN_3_) was used for the permeabilization of the samples containing 0.1% of saponin. The nucleated cells were labeled with DAPI for 30 min (Tempone et al., 2010). The analyses of the samples were made in a fluorescent microscopy Nikon Eclipse 80i-D-FL-EPI.

### Statistical Analysis

The data obtained were reported as the mean and standard deviation of duplicate samples from two or three independent assays. The data were analyzed using GraphPad Prism 5.0 software, using one-way ANOVA for significance testing (*P* < 0.05). For the immunomodulatory studies, the data were analyzed using the non-parametric *t-*test (*P* < 0.05).

## Results

### Determination of the *in vitro* 50% Effective Concentration (EC_50_) and 50% Cytotoxic Concentration (CC_50_)

SERT and the standard drug miltefosine were incubated with promastigotes of *L*. (L.) *infantum* and the viability was determined by the MTT assay. After 48 h of incubation, the drugs showed EC_50_ values of 0.7 and 16 μM, respectively (Table 1). SERT was also effective against intracellular amastigotes and showed an EC_50_ value of 4.2 μM. The liposomal formulation of SERT (LP-SERT) and miltefosine showed EC_50_ values of 2.5 μM and EC_50_ 3.1 μM, respectively. The mammalian cytotoxicity was evaluated using NCTC cells after 72 h of incubation, and the viability was determined by MTT assay. The free and liposomal drug (SERT and LP-SERT) showed CC_50_ values of 27.4 and 12.0 μM, respectively (Table 1).

**Table 1:** *In vitro* 50% effective concentration (EC_50_) of free and liposomal sertraline against *L*. (L.) *infantum* and mammalian cytotoxicity.

**EC**_****50****_ **(μM)** ± **SD**				
**Drugs**	***L***. **(L)** ***infantum***** promastigotes**	***L***. **(L)** ***infantum***** amastigotes**	**Cytotoxicity NCTC cells**	**S.I**.
SERT	0.7 ± 0.4	4.2 ± 2.2	27.4 ± 0.8	6.4
LP-SERT	–	2.5 ± 1.6	12.0 ± 2.4	4.8
Miltefosine	16.2 ± 0.02	3.1 ± 1.6	127.7 ± 6.7	40.9

*EC_50_, 50% effective concentration; S.I., selectivity index [CC_50_ mammalian cells/EC_50_ L. (L) infantum amastigotes]; SERT, free sertraline; LP-SERT, liposomal sertraline; SD, standard deviation; EC_50_ values are representative of three independent assays*.

### Internalization of LP-SERT-R123 in Peritoneal Macrophages

**Figure 1:**
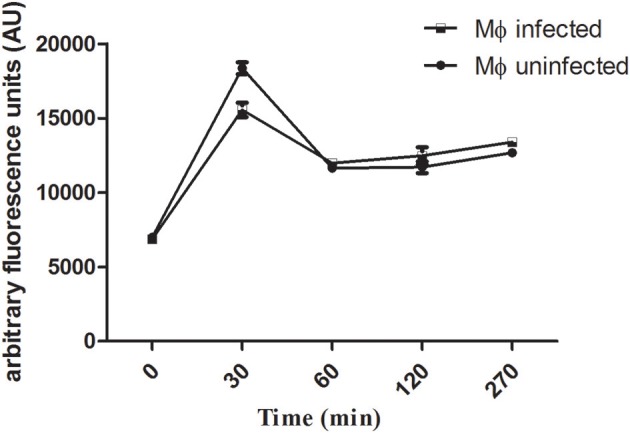
Uptake of liposomal sertraline labeled with rhodamine 123 by *Leishmania*-infected and uninfected macrophages. The uptake was measured by the fluorescence intensity within macrophages using a flow cytometry (Attune-Thermofisher) for a period of 270 min. A number of 10,000 events were analyzed for each time.

The uptake capacity of *Leishmania*-infected macrophages and uninfected macrophages to internalize rhodamine 123-labeled liposomes was analyzed by flow cytometry. The peak of internalization of LP-SERT-R123 occurred within the first 30 min for both groups (infected and uninfected macrophages), with similar levels of fluorescence. At 60 min, the fluorescence levels dropped and remained sustained in both groups of macrophages until 270 min (Figure 1).

### Physico-Chemical Characterizations of LP-SERT

In order to characterize the influence of SERT in PS-containing liposomes, DLS and ζ-potential experiments were performed. DLS measurements indicated that SERT was not able to alter the overall size of the liposomes, which showed an average diameter (*Z*-average) of ~127 nm in the absence and 128 nm in the presence of SERT, respectively. PDI values were also calculated and remain 0.27 and 0.33 in the absence and presence of SERT, respectively. The liposome ζ-potential was also unchanged after the entrapment of SERT, which showed a negatively charged formulation of −64 ± 4 mV in the absence and −58 ± 5 mV in the presence of SERT. Taken together, both DLS and ζ-potential measurements indicated that the SERT was not able to change significantly either the vesicle effective diameter or its superficial charge. The quantification of SERT in liposomes was determined by HPLC, using a standard curve of SERT. The Encapsulation Efficiency (%EE), resulted in a mean value of 80% ± 3%.

### *In vivo* Efficacy of SERT

**Figure 2:**
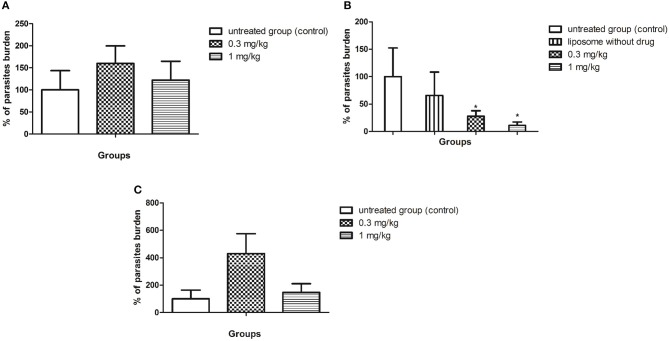
*In vivo* efficacy of SERT. After 5 days of infection with *L*. (L.) *infantum*, BALB/c mice were treated for 10 consecutive days. **(A)** Parasite burden (%) of the treatment with free sertraline in oral administration. **(B)** Parasite burden (%) of the treatment with liposomal sertraline in subcutaneous administration. **(C)** Parasite burden (%) of the treatment with free sertraline in subcutaneous administration. ^*^p < 0.05.

BALB/c mice were infected with *L*. (L.) *infantum* (1 × 10^8^ promastigotes) and after 5 d.p.i. the animals were treated for 10 consecutive days with SERT by oral route at 0.3 and 1 mg/kg. The culture microtitration analysis demonstrated that the treatment with SERT was not able to reduce the parasite burden when compared with the untreated group (Figure 2A). Considering the therapeutic failure of SERT, the drug was entrapped into negatively charged liposomes and administered by subcutaneous route using the same doses. The results indicated that the treatment with LP-SERT was able to reduce the liver parasite burden by 72% at 0.3 mg/kg (*P* < 0.05) and by 89% at 1 mg/kg (*P* < 0.05) when compared with the untreated group. Free liposomes (without SERT) were used as a control and demonstrated no efficacy (Figure 2B). The efficacy of free SERT (unentrapped) was also evaluated by subcutaneous route and used as an internal control. The free drug was not able to eliminate the parasites in liver when administered by the subcutaneous route (Figure 2C). All animals survived after the treatment with SERT and LP-SERT. During the time of infection (15 days), 100% of animals survived in the untreated group.

### *In vivo* Immunomodulatory Effect of LP-SERT

**Figure 3:**
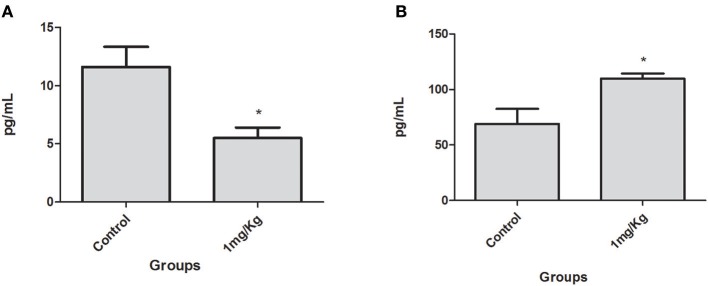
Serum IL-6 **(A)** and chemokine MCP-1 **(B)** levels in BALB/c mice infected with *L*. (L.) *infantum* treated for 10 consecutive days with LP-SERT at a dose of 1 mg/kg. Cytokine levels (pg/ml) were determined by flow cytometry by the CBA kit (cytometric beads array—BD) (^*^p < 0.05). It was considered 10,000 events per analysis. The control is defined by infected and untreated animals.

After 10 consecutive days of treatment with LP-SERT, the serum was collected and the cytokine levels of *L*. (L.) *infantum-*infected BALB/c mice were evaluated by flow cytometry. The results demonstrated a significant downregulation (two-fold) of the interleukin 6 (IL-6) levels of the group treated at 1 mg/kg. The chemokine MCP-1 levels were also significant upregulated in the treated group (Figure 3). Other cytokines showed undetectable levels in serum.

### Histopathology Profile of BALB/c Mice Treated With LP-SERT

**Figure 4:**
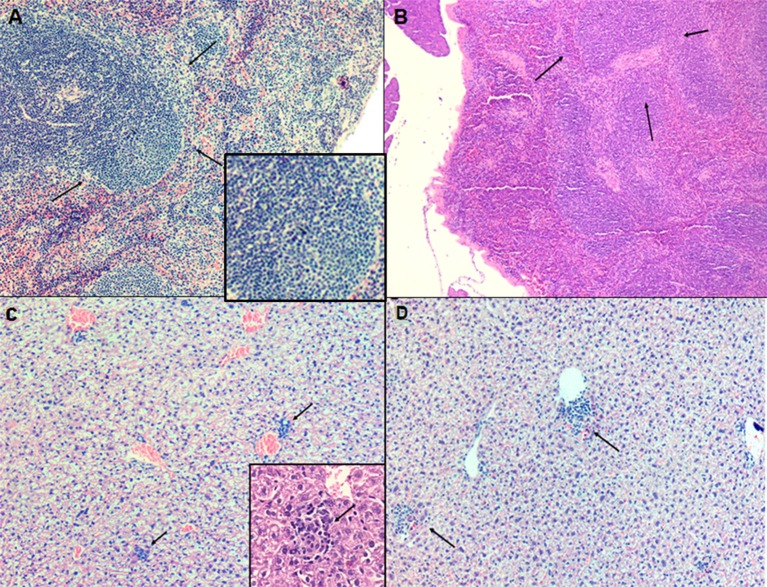
Evaluation of histopathological profile of BALB c mice infected with *L*. (L.) *infantum*, after 10 consecutive days of treatment with liposomal sertraline. **(A)** Spleen of an animal representative of untreated group. **(B)** Spleen of an animal representative of the group treated with liposomal sertraline at 1 mg/kg. **(C)** Liver of an animal representative of the untreated control group. **(D)** Liver of an animal representative of the group treated with liposomal sertraline at 1 mg/kg. Presence of granulomas in all groups (arrows). Staining of H&E, 100× magnification.

The spleen and the liver of untreated and animals treated (LP-SERT at 1 mg/kg) were evaluated using histopathological techniques. The light microscopy analysis of the slides demonstrated no significant differences between untreated infected animals and LP-SERT-treated groups. We observed the presence of a lymphoid hyperplasia in the spleen (Figures 4A,B) as well as an inflammatory infiltrate with the development of granulomas in liver (Figures 4C,D).

### Targeting of LP-SERT to Spleen and Liver

**Figure 5:**
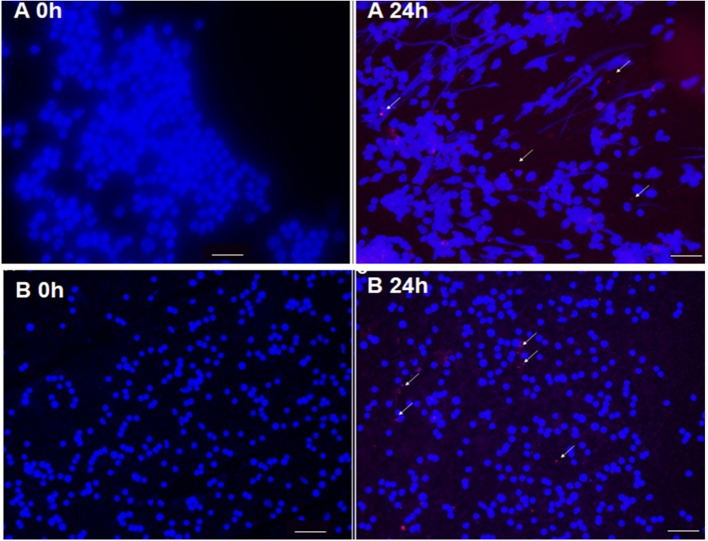
Targeting of liposomes (arrow) labeled with DIL C18 (red) containing sertraline in spleen **(A)** and liver **(B)** of BALB/c mice. Ten days after infection, the animals were treated with LP-SERT, spleen and liver were removed, imprinting of each organ have been made in slides, and the samples were submitted to immunofluorescence analyses at 24 h. Nucleated cells were labeled with DAPI (blue). White bars represent 50 μm.

The liposomal formulation of SERT was labeled with the fluorescent probe DIL C18 and administrated by subcutaneous route in infected *L*. (L.) *infantum* BALB/c mice. The fluorescence microscopy analyses demonstrated that LP-SERT was distributed to the liver and spleen of the infected animals after 24 h of administration (Figure 5).

## Discussion

VL affects millions of poor people worldwide and remains without safe therapeutic options; drug repurposing or repositioning has been an important approach, reducing time and cost of the research. Previous studies demonstrated that antidepressants like sertraline and others showed anti-*Leishmania* activity (Palit and Ali, 2008; Mukherjee et al., 2012). Palit and Ali (2008) have demonstrated that SERT eliminated 72 and 70% of parasite burden of spleen and liver, respectively, using a *L. donovani*-infected BALB/c mice model. SERT is a serotonin reuptake inhibitor approved by the Food and Drug Administration (FDA), and it is widely used in the treatment of depression (Santuzzi et al., 2012; Onaolapo et al., 2017). In this study, the therapeutic potential of SERT and LP-SERT against *L*. (L.) *infantum* was determined using *in vitro* and *in vivo* models.

Our studies demonstrated that SERT was 22 times more effective against promastigotes than the standard drug miltefosine. SERT was also able to eliminate 100% of intracellular amastigotes with similar activity to miltefosine, resulting in an SI of 6. Palit and Ali (2008) previously showed that SERT was effective against *L*. (L.) *donovani*, with EC_50_ values 1.6- and 10-fold smaller than our studies with *L*. (L.) *infantum* against promastigotes and intracellular amastigotes, respectively. Although *L*. (L.) *donovani* is also a viscerotropic species, the differences found in our studies could be ascribed to the differences in the species between *L*. (L.) *infantum* and *L*. (L.) *donovani*, resulting in different drug susceptibilities (Costa-Silva et al., 2015). Genetic markers for sensibility to specific drugs have been identified in viscerotropic *Leishmania* species, specifically *L*. (L.) *donovani* and *L*. (L.) *infantum*. The presence of Miltefosine Sensitivity Locus (MSL) in DNA resulted in a susceptible pattern to miltefosine, also modifying the clinical responsiveness to the drug. *Leishmania* parasites lacking MSL were shown to be resistant to miltefosine (Carnielli et al., 2018). Additionally, differences in DNA fingerprint analyses have been identified for *L*. (L.) *donovani* and *L*. (L.) *infantum* (Ellis and Crampton, 1991). Future genetic studies using *L*. (L.) *donovani* and *L*. (L.) *infantum* could demonstrate specific genetic markers that contribute to the response to sertraline.

The use of FDA-approved drugs for drug repositioning approach has considerable advantages, especially when one considers the available ADMET data of individual drugs. Data from the literature report that sertraline has a plasma half-life (*T*_1/2_) of 8 h when orally administered in healthy patients, and more than 26 h for the plasma terminal *T*_1/2_ (Murdoch and McTavish, 1992). Considering the *in vitro* potency of sertraline against *L*. (L.) *infantum*, we conducted an *in vivo* efficacy study in a murine model, using the following approach: (i) administration of SERT by oral route; (ii) administration of a daily single dose for 10 consecutive days; (iii) considering the HED index to translate doses human↔animal (Reagan-Shaw et al., 2008), a dose of 1 mg/kg was chosen based on the human equivalent dose of 0.1 mg/kg; a maximum human dose of sertraline as an antidepressant drug is 3 mg/kg, but this is followed by significant increase in adverse effects (Drugbank, 2017). This murine visceral model of Leishmaniasis has been useful for the evaluation of drug candidates. Cunha-Junior et al. evaluated the time course of the infection using the same strain of *L*. (L.) *infantum* in BALB/c mouse. After intra-peritoneal infection with 1 × 10^8^ stationary-phase promastigotes, the parasite burden was analyzed by the microtitration method at days 7, 14, 21, and 30. The infection was well-established after 7 days, with parasites in the spleen and liver, with increased and sustained infection for 30 days. Our *in vivo* assays of oral SERT showed no reduction of the parasite burden. This result could be ascribed to multiple factors, including a low oral absorption of SERT, a poor delivery of SERT, resulting in low tissue concentration of the drug in the spleen and liver, or the metabolization of SERT, which could have generated an inactive metabolite against *L*. (L.) *infantum*. Ronfeld et al. (1997) demonstrated that sertraline has low oral absorption in young human volunteers, with a *T*_max_ of 7 h. The authors also report a low absorption for sertraline after oral administration with a *C*_max_ value of 118 ng/ml. Additionally, data from DrugBank (https://www.drugbank.ca/drugs/DB01104) report that sertraline administered once daily at 50–200 mg for 14 days resulted in steady-state concentrations after 1 week. Additionally, the drug is extensively metabolized by the liver to form *N*-desmethylsertraline, which has a lower pharmacological antidepressant effect than the parent compound.

Considering the therapeutic failure of free SERT, we performed the entrapment of the drug into negatively charged liposomes containing phosphatidylserine. Sertraline demonstrated a high entrapment (82%) into the formulation, resulting in the formation of nanovesicles covered by a negative charge. Drug delivery systems (DDS) as liposomes have been widely used in the pharmaceutical field (Kansal et al., 2014). In the clinical therapy of leishmaniases, DDS have been used for many years, as the liposomal amphotericin B (Ambisome), which provides reduced doses, higher safety, and increased therapeutic index (Ostrosky-Zeichner et al., 2003). In this work, we observed that the encapsulation of the SERT in negatively charged liposomes resulted in an *in vivo* efficacy of the drug, reducing 72% of the parasitic burden in the liver at doses below 1 mg/kg. At this dose (1 mg/kg), the parasite burden was reduced by 89% in the liver. In a previous study, miltefosine was administered to a BALB/c model infected with *L*. (L.) *infantum* (MHOM/MA67ITMAP263) and resulted in 100% suppression of the parasite burden in liver and spleen at 7.7 mg/kg (Rebello et al., 2019).

VL causes strong alterations in the immune system; Ansari et al. (2006) observed the serum of VL-patients and reported an increase of the cytokine IL-6, which was associated to the progressive form of the disease. Murray (2008) studied IL-6 knockout mice and observed a better outcome of animals treated with pentavalent antimonial and amphotericin B when compared to IL-6 competent mice. In our studies, significant differences of IL-6 levels were observed between treated and untreated groups. Additionally, the chemokine MCP-1 was analyzed, and we observed increased serum levels in mice treated with LP-SERT at 1 mg/kg. Chemokines constitute a family of chemoattractant cytokines and play a major role in selectively recruiting monocytes, neutrophils, and lymphocytes, as well as in inducing chemotaxis through the activation of G-protein-coupled receptors. Monocyte chemoattractant protein-1 (MCP-1/CCL2) is one of the key chemokines that regulate migration and infiltration of monocytes/macrophages (Deshmane et al., 2009). This chemokine is produced by macrophages, dendritic cells, and monocytes, and it is an important chemotactic factor for T cells and natural killer cells (Deshmane et al., 2009) for the formation of granulomas in the liver, promoting the extermination of the parasites (Cotterell et al., 1999; Dey et al., 2007). Our histopathological profile showed an increase of defense cells as macrophages and dendritic cells, besides the presence of hepatic granuloma and lymphoid hyperplasia in the spleen. This fact could be ascribed to a possible strategy of the organism to activate a response against the parasite after sertraline exposure. Additionally, these histopathological data corroborate the increased serum levels of the monocyte chemoattractant protein (MCP-1) and suggest the ability of LP-SERT to modulate the immune response of animals, eliminating *L*. (L.) *infantum* in liver. Although LP-SERT was able to reduce the parasite burden after a course of 10 days of treatment, the presence of hepatic granuloma and lymphoid hyperplasia demonstrates an active and unsolved inflammation. Future studies with higher courses (>10 days) of LP-SERT administration could contribute to tissue regeneration. Sertraline was also administered as a free formulation by the subcutaneous route. Our results demonstrate that free SERT was not able to reduce the parasite burden at any tested doses.

The physicochemical properties of liposomes drive the targeting ability of the formulation, and among them, the membrane charge and the vesicle size are critical issues. Negatively charged liposomes containing phosphatidylserine result in a higher uptake by macrophages via scavenger receptors (SRs), with an increased *in vitro* efficacy against *Leishmania* (Tempone et al., 2004), and the VL-mice model (Laverman et al., 1999; Mukherjee et al., 2012). The negative charge of vesicles (the modulus of the ζ-potential values >30 mV) also prevents aggregation of the vesicles, avoiding destabilization of the formulation (Walton et al., 2010). Another important characteristic is the vesicle size; large liposomes (>400 nm) are rapidly removed from the circulation when compared to smaller liposomes (<400 nm) (Faleiro et al., 2014). Considering that *Leishmania* is located inside the mononuclear monocyte system in spleen, liver, bone marrow, and lymph node (Lindoso et al., 2016), a formulation composed of nanostructures is essential. Reimão et al. (2012) demonstrated that liposomes smaller than 200 nm were able to reduce 80% of the parasite burden in bone marrow of *L*. (L.) *infantum*-infected hamsters.

The route of administration directly defines the therapeutic success of a drug. Previous studies of our group demonstrated that the subcutaneous route can increase the efficacy of negatively charged liposomes containing buparvaquone in an *in vivo* VL-model (Costa-Silva et al., 2017). Considering the efficacy of LP-SERT to reduce the parasite burden in liver of BALB/c mice, we studied the targetability of this formulation labeled with a fluorescent probe. After 24 h of the administration, it was possible to observe the presence of LP-SERT in the liver and spleen of previously infected animals. These data demonstrated that LP-SERT reached the *Leishmania* target organs, releasing the drug at or nearly the host cells when administrated subcutaneously. Additionally, our *in vitro* studies with infected macrophages demonstrated that LP-SERT-R123 was able to internalize into *Leishmania*-infected and uninfected macrophages at similar levels. Dhanikula et al. (2005) demonstrated that the pharmacokinetics parameters of the liposomal formulation of paclitaxel were widely distinct when administrated subcutaneously and intravenously; the drug bioavailability was improved by the subcutaneous route. Allen et al. (1993) observed the administration of liposomes in different routes (subcutaneous, intravenous, and intra-peritoneal); their results showed undamaged liposomes in the bloodstream after passing for the lymphoid chain when administrated via the subcutaneous route. In our previous studies with liposomal buparvaquone, the subcutaneous route was also the best option to improve the drug efficacy and targeting ability.

## Conclusion

The present study demonstrated that sertraline is a potent *in vitro* anti-*L*. (L.) *infantum* drug. For the first time in the literature, sertraline was encapsulated in phophatidylserine liposomes and studied in an experimental VL-murine model. The liposomal formulation of sertraline demonstrated the ability to reduce the parasite burden at low doses, being delivered to the liver and spleen of infected mice. The study also demonstrated that at higher doses, the liposomal sertraline can modulate the host immune response, affecting the course of the disease. Future pharmacokinetic studies may contribute to understand the action of this new formulation in VL.

## Data Availability Statement

The datasets generated for this study are available on request to the corresponding author.

## Ethics Statement

Animal experiments were performed with the approval of the Ethics Committee of Instituto Adolfo Lutz (project CEUA-IAL/Pasteur 04/2016) in accordance with the National Institutes of Health guide for the care and use of Laboratory animals (NIH Publications No. 8023).

## Author Contributions

MR developed all experimental work. TC-S and DD developed part of the *in vitro* studies with sertraline. JG performed histopathology studies in animals. AG performed part of flow cytometry studies for cytokine detection. EC-J standardized animal studies with Leishmania. EP performed initial studies with sertraline in animals. ET-S standardized animal studies with Leishmania. LB performed physico-chemical characterization of liposomes. AT was responsible for the design and general idea of the work and analysis of *in vitro* and *in vivo* studies.

### Conflict of Interest

The authors declare that the research was conducted in the absence of any commercial or financial relationships that could be construed as a potential conflict of interest.
